# Preventing soft skill decay among early-career women in STEM during COVID-19: Evidence from a longitudinal intervention

**DOI:** 10.1073/pnas.2123105119

**Published:** 2022-08-01

**Authors:** Julia L. Melin, Shelley J. Correll

**Affiliations:** ^a^Department of Sociology, Stanford University, Stanford, CA 94305;; ^b^VMware Women's Leadership Innovation Lab, Stanford University, Stanford, CA 94305

**Keywords:** gender, soft skills, COVID-19, hybrid and remote work, online interventions

## Abstract

As the workforce shifts to being predominantly hybrid and remote, how can companies help employees—particularly early-career women in science, technology, engineering, and mathematics (STEM) fields—develop greater confidence in their soft skills, shown to improve organizational retention? We evaluate the effects of an online longitudinal intervention to develop soft skills among early-career women employees at a North American biotechnology company during the height of the COVID-19 pandemic. Controlling for baseline levels collected immediately prior to nationwide lockdowns, we find that a 6-month online intervention increased early-career women’s assessments of their soft skills at work by an average of 9% (*P* < 0.001), compared with a decrease of about 3.5% for a matched control group (*P* < 0.05), resulting in an average treatment effect of nearly 13% on the treated group. Furthermore, we find evidence that the intervention led to an increase in manager-assessed performance for early-career women relative to employees not in the intervention, and that overall, increased self-assessments of soft skill competencies were associated with greater odds of retention. Results show how employee soft skill development was affected by the pandemic and provide insights for a feasible and cost-effective method to train and engage a hybrid or fully remote workforce.

## Significance

Women leave science, technology, engineering, and mathematics (STEM) fields at higher rates than men do. The COVID-19 pandemic has exacerbated this problem. As companies emerge from the pandemic, interventions that prevent the gender gap from widening are critical for retaining a diverse STEM workforce. We evaluate an intervention to improve women’s confidence in their soft skills, an important predictor of workplace retention among women. We leverage rare longitudinal data collected from biotechnology employees immediately before and during the pandemic. Early-career women in the intervention experienced significant gains in their perceived soft skills, while similarly situated women experienced a decline. Furthermore, soft skill improvements were associated with significant increases in retention, suggesting the importance of soft skill development for early-career women post-pandemic.

Employers have long struggled to effectively train their workers on “soft skills,” broadly defined as abilities and behaviors that allow employees to work well with others (1, 2). There is a large and growing literature on soft skills, a topic that generally focuses on how interpersonal skills, such as teamwork, communication, resilience, and the ability to influence and build strategic networks, along with other *interpersonal-related* abilities, compare with “hard skills” (e.g., technical, cognitive, and other *achievement-related* abilities) (1–8).* While studies suggest that soft skills have become more important than hard skills in predicting career success (3, 7), these types of skills remain elusive among prospective job applicants (6, 9). In fact, today’s employers consistently cite teamwork, collaboration, and communication skills as highly desirable yet rare qualities among applicants (7).

In this article, we analyze whether an online longitudinal intervention conducted in a biotechnology firm improved early-career women employees’ self-assessments of their soft skill competencies (which we also refer to as “perceived soft skills"). We focus on self-assessments for two main reasons. First, there is evidence that self-assessments related to confidence have meaningful consequences for individual career outcomes, particularly with respect to persistence and retention (10–16)—a finding we also confirm in this article. Research has shown that individuals who possess high core self-evaluations (e.g., high self-esteem and generalized self-efficacy) are more likely to persist on difficult tasks and stay motivated despite rejection or failure (15). In contrast, individuals with low core self-evaluations are not only more likely to quit their jobs when they become difficult but they may also self-sabotage their progress by quitting as circumstances begin to improve (15, 16). Second, gender differences in self-assessments have been shown to contribute to the gender gap in science, technology, engineering, and mathematics (STEM) (11–14, 17). For instance, women make lower assessments of their own mathematical ability and report lower levels of professional-role confidence in STEM compared with men, which contributes to women’s overall attrition from STEM fields, net of their actual performance (11, 13, 14).

For women in STEM, leveraging soft skills for professional advancement is especially difficult due to persistent structural and social barriers (11, 12, 18, 19). Although certain soft skills (e.g., listening to and caring for others) are typically attributed to women, these communal soft skills are not sufficient to get ahead in the workplace (20). Instead, the ability to influence colleagues and build strong operational and strategic networks are the high-status soft skills most necessary yet hardest to achieve for women in male-dominated fields (18, 21). These barriers contribute to early-career women leaving STEM at higher rates than men and women in other fields (22, 23).

The COVID-19 pandemic likely exacerbated these challenges, when most professional organizations abruptly transitioned their employees into being a fully remote workforce. Women experienced disproportionate career setbacks, either downshifting their responsibilities or leaving their jobs entirely, primarily due to increased childcare obligations when schooling became remote (24–26). Now that a large share of the professional workforce plans to operate on a hybrid or fully remote basis indefinitely (27), employers must develop new methods to help employees cultivate the soft skills required for building effective operational and strategic networks virtually (28, 29). While some have expressed optimism about the long-term benefits of hybrid and remote work for women balancing careers and families (30), others remain concerned that women’s greater preference for hybrid and remote work, compared with that of men (27), could exacerbate gender inequality (31). Programs designed to enhance soft skill development will likely be important for women—especially early-career women—to level the playing field in this new world of hybrid and remote work.

In November 2018, we started a research collaboration with a digital “talent experience platform” (herein, TXP)—an increasingly common type of software offering designed to enhance the overall employee experience (e.g., by fostering a sense of belonging and purpose at work) (32). Our research objective was to study how online career coaching and virtual peer groups offered on such platforms affect women’s self-assessments of their ability to navigate interpersonal relationships at work, since confidence in one’s abilities is strongly linked with persistence and retention (10–16). The TXP was interested in testing their platform’s offerings among early-career women—those most at risk for attrition from STEM and other male-dominated fields (22, 23). A year later, we began a three-way partnership with a large North American biotechnology company to conduct the intervention with their employees. During this time, we did not anticipate how much the world would change in the coming months and how relevant our online study would be for a fully remote workforce during a pandemic.

Our research scope began with the question “How does an online intervention program (consisting of virtual peer groups and online career coaching) affect confidence in soft skill acquisition among early-career women?” We collected baseline measures from a sample of men and women employees at the company between January and February 2020, just prior to nationwide lockdowns. Because our intervention began shortly after nationwide lockdowns, we wondered if our online intervention would buffer against some of the negative effects that women employees generally reported experiencing at the time (24, 25). Our timing and the online nature of our intervention allowed us to keep our study design intact while simultaneously evaluating our results in the context of the pandemic. We treated COVID-19 as a natural experiment and expanded our research scope to ask: “Did early-career women experience soft skill decay during the pandemic, and if so, did our intervention produce a buffering effect against this decay?” We used longitudinal survey and administrative data to evaluate how the pandemic affected employees’ sense of soft skill development, and whether the online intervention led to different outcomes for early-career women in the intervention compared with men and women who did not undergo the intervention.

The mass social experiment of working from home during the pandemic has largely been viewed as a success due to increased productivity levels (27, 33, 34). However, others have expressed concerns about the long-term sustainability of this model, given the loss of in-person interactions, which many executives and managers feel are critical for building high-performing teams and a strong organizational culture (34, 35). Prior to COVID-19, research examining the effects of telework had shown somewhat mixed results. While some studies found a negative relationship between time spent teleworking and individual and team performance (36, 37), other experimental work provided causal evidence showing the opposite, with performance significantly boosted among willing workers randomly assigned to work from home versus in the office for 9 months (38). Another study comparing virtual teams with colocated teams found that soft skills (e.g., strong communication and interpersonal coordination) are a major prerequisite for team performance and, therefore, should be carefully considered by managers when assembling virtual teams (28).

While we are not aware of any studies directly assessing the effect of telework on the development of soft skills, it is likely that soft skill development is more challenging in a remote work environment, making it harder, for example, to develop strong social networks (37, 39). These challenges will likely persist in a post–COVID-19 world, despite the recent universal adoption of online communication technology (e.g., Zoom, Microsoft Teams, Slack). In fact, some have raised the concern that hybrid working arrangements will be the worst of both worlds, noting that when all employees worked fully remote, at least it was a level playing field (40, 41). If women are more likely to prefer remote work in a hybrid organization (27), women could be further disadvantaged, especially those who are early career and have less experience building the networks required for organizational advancement (11, 12). Since women shoulder the majority of caregiving responsibilities, they may be more likely than men to opt for at-home work arrangements and miss out on in-person networking that often leads to promotion (31).

Our results show that remote work during the COVID-19 pandemic negatively affected self-assessments of soft skill development among early-career women who were not in the intervention but did not affect these skills for men employees or women more advanced in their careers. Most importantly, we found that the online intervention not only buffered against soft skill decay for early-career women during the pandemic but even enhanced their perceived acquisition of these skills relative to early-career women who were not in the intervention. We also found that our intervention had positive implications for both manager-assessed performance and employee retention.

## Results

Table 1 presents *t-*tests comparing pre- and post-intervention means within each condition and post-intervention means between conditions. Women who received the online intervention reported an increase in perceived soft skills by an average of 0.32 points on a 5-point scale (*P* < 0.001), a 9.12% increase, while women in the matched control group who did not receive the intervention reported a *decrease* in perceived soft skills by an average of 0.13 (*P* < 0.05), a 3.52% decline over the same period. Men and women in the pooled control group reported a nonsignificant increase of 0.04, or 1.04%, in perceived soft skills. When comparing across conditions, we found that the post-intervention mean for women who received the intervention was significantly higher than that of women in the matched control group (*P* < 0.05). We found no significant difference in post-intervention means between the treatment group and the pooled control group.

**Table 1.** Means and *P* values from two-sided *t-*tests comparing pre- and post-intervention means of employee-perceived soft skills by condition

	Pre-intervention mean	Post-intervention mean	Mean difference*	*P* value	Post-intervention *P* value of T vs. C_M_	Post-intervention *P* value of T vs. C_P_
T	3.538 (0.509)	3.861 (0.480)	0.323 (0.064)	<0.001		
C_M_	3.740 (0.516)	3.607 (0.488)	−0.132 (0.053)	0.018	0.033	
C_P_	3.845 (0.522)	3.886 (0.488)	0.040 (0.034)	0.255		0.806
*N*	148	148				

Values are presented as mean (±SD) for group means and mean (±SE) for pre–post group differences. The perceived soft skills factor variable is measured using a 5-point scale. C_M_, matched control; C_P_, pooled control; T, online intervention.

^*^Post-intervention minus pre-intervention.

The results were slightly stronger when re-estimated using an analysis of covariance (ANCOVA) model adjusted for baseline scores, age, employee tenure, and compensation level. In Table 2, model 2 shows the effect of the online intervention (the reference category) on perceived soft skills post-intervention compared with the results for the matched control and pooled control. After adjusting for baseline differences, we now find that the intervention led to significantly higher self-assessments of soft skill development for women in the intervention compared with employees in both control groups. Compared with women who received the intervention, women in the matched control group reported significantly lower perceived soft skills following the intervention period ($\hat{\beta }$ = −0.424; *P* < 0.001). We also observe that men and women in the pooled control group reported significantly lower perceived soft skills compared with women in the treatment group following the intervention period ($\hat{\beta }$ = −0.283; *P* < 0.01).

**Table 2.** Analysis of covariance estimates of the effect of condition type on employee-perceived soft skills post-intervention

	Model 1	Model 2	Model 3
Condition type (Reference = online intervention)			
Matched control	−0.404*** (0.075)	−0.424*** (0.081)	0.167 (0.132)
Pooled control	−0.204** (0.067)	−0.283** (0.098)	0.192 (0.143)
Perceived soft skills (baseline score)	0.740*** (0.048)	0.724*** (0.048)	
Condition type × post-intervention score			
Online intervention			0.323*** (0.064)
Matched control			−0.132* (0.054)
Pooled control			0.040 (0.035)
Age		0.008* (0.004)	0.012 (0.006)
Tenure		−0.009 (0.005)	−0.018 (0.010)
Compensation grade		−0.005 (0.015)	−0.004 (0.025)
Constant	1.243*** (0.184)	1.111*** (0.191)	3.240*** (0.183)
Observations	296	296	296
*R*^2^ value	0.642	0.655	0.096

Robust SEs are in parentheses. Observations were clustered at the employee level. *P < 0.05, **P < 0.01, ***P < 0.001.

To assess the adjusted within-group changes, model 3 includes an interaction term between each of the three groups and a dummy indicator for perceived soft skill scores following the intervention period (for more information on this approach, see ref. 42). Results confirmed that women who received the intervention reported a significant increase in perceived soft skills from baseline ($\hat{\beta }$ = 0.323; *P* < 0.001), whereas women in the matched control group reported a significant decrease on the same measure following the intervention period ($\hat{\beta }$ = −0.132, *P* < 0.05). Men and women in the pooled control group did not experience a significant change from baseline.

**Fig. 1.**
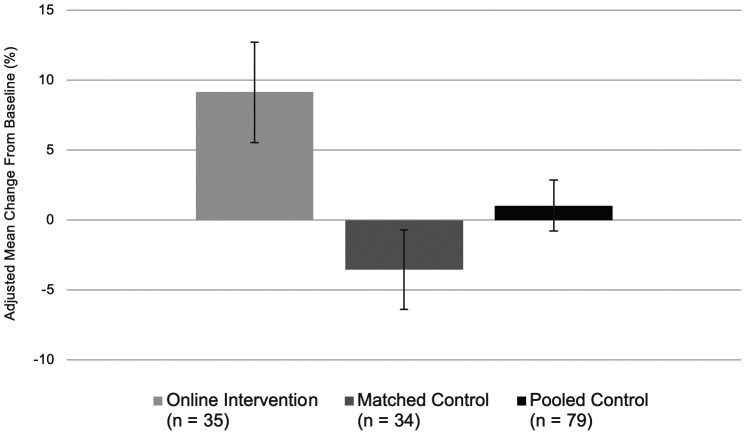
Adjusted mean percentage change in employee-perceived soft skills from pre–COVID-19 baseline levels. *N* = 148. Bars show 95% CIs. Perceived soft skills measure represents the average across the six-item scale (see Table 3).

Fig. 1 illustrates our main results by showing the percentage change from baseline by experimental condition. Bar graphs represent results converted to a percentage change using mean baseline and post-intervention scores (for more information on this approach, see ref. 43). Adjusting for baseline differences, we find similar results to those reported in the preceding paragraphs: Women in the online intervention group experienced a significant increase in perceived soft skills by 9.15% from pre–COVID-19 baseline levels, while women in the matched control group experienced a significant decrease of 3.53% in perceived soft skills over the same period. Men and women in the pooled control group experienced a nonsignificant increase of 1.04% from baseline.

Taken together, these findings suggest that the average treatment effect on the treated group was 12.68%, implying that if women in the treatment group had not received the intervention, their perceived soft skills would be reduced by nearly 13% relative to their post-intervention level. Put differently, each month of the 6-month program contributed to an additional 2% boost in perceived soft skills over the course of the intervention period for early-career women.

While our primary focus was on whether our intervention increased women’s assessments of their soft skills, we also examined additional administrative data provided by the company and found that the intervention led to a significantly higher increase in managers’ performance ratings for early-career women in the intervention relative to those in both control groups (*SI Appendix*, Table S2, model 2).

Furthermore, and consistent with prior research showing that self-assessments related to professional confidence are consequential for retention (10–16), we found that overall (i.e., across the sample of study participants) and holding constant age, tenure, and compensation, employees who had higher soft skill self-assessments following the intervention period had significantly higher odds of still being employed at the company 1-year post-intervention (*SI Appendix*, Table S3, model 2). By contrast, manager-assessed performance did not predict employee retention (*SI Appendix*, Table S4, model 2). These additional findings suggest that employees’ assessments of their own interpersonal abilities at work are more predictive of organizational retention than the assessments made by their managers.

## Discussion

As companies attempt to figure out a post-pandemic working model, many will adopt hybrid or remote work schedules for their employees. Given the recent disproportionate attrition of women from the labor market (24, 25, 44), interventions that prevent the gender gap from widening are critical for retaining and developing a diverse workforce, particularly in STEM and other male-dominated professions. Going forward, organizations will need to develop new methods to help employees—particularly newly hired employees—cultivate the confidence in their soft skills necessary for retention and long-term success.

Overall, we found a decay in soft skills among early-career women in STEM working remotely during the COVID-19 pandemic. Examining longitudinal data collected from employees at a large US biotechnology company, we found that early-career women reported a significant decline in perceived soft skills by 3.5% from pre–COVID-19 baseline levels. Yet early-career women who participated in an online intervention program consisting of peer groups and one-on-one coaching were not only shielded from soft skill decay, they also experienced a growth in their perceived soft skills by more than 9% during the same period, resulting in an average treatment effect of nearly 13% on the treated group. We further found that the intervention was associated with employees receiving higher performance evaluations from their managers, while also having important implications for employee retention.

Because our study did not involve randomization to treatment and control conditions, causal inferences should be interpreted with caution. Nonetheless, these data offer rare pre- and post–COVID-19 pandemic insights into how the pandemic affected STEM employees as the pandemic evolved. These findings also offer potential solutions for preventing soft skill atrophy and developing soft skills via a virtual format. Moreover, while this study was conducted within a single organization, results are likely generalizable to other organizations, given that the workplace challenges at the height of the pandemic were not unique to the organization in this study. While the present research examined how an online longitudinal intervention buffered against the negative effects of COVID-19, it is unclear how such an intervention would perform in a nonpandemic, remote environment. For instance, without the added personal stress and organizational disruption produced by the pandemic, it is possible that this program would have had an even greater positive effect on early-career women working remotely. Regardless, our online intervention produced positive effects despite not being specifically designed for a remote working environment. This outcome suggests that soft skill development is not just important for in-person work but is also valuable for remote work. Given major concerns among executives and managers that remote work will cause individual and team job performance to suffer (34, 35), companies planning to maintain a remote or hybrid workforce would likely benefit from similar interventions focused on enhancing the soft skills of their employees.

## Experimental Design

In December 2019, the study received approval from the Institutional Review Board at Stanford University. Study recruitment took place between January and February 2020. All participants provided informed consent.

For participants in the online intervention (herein, the treatment group), we worked with the biotechnology company’s human resources analytics team to randomly identify 44 early-career women who were under the age of 33 and possessed less than 10 years of work experience. The company’s analytics team used manager performance data to ensure that there was not an overrepresentation of high-performing women recruited for the intervention. Managers of potential participants were contacted about the study and asked to support and encourage those women who wished to participate.

Upon recruitment, the study was broadly positioned as an opportunity to receive the following: 1) a virtual peer-support network intended to foster connections with other women in the organization at similar stages; 2) the chance to receive hands-on guidance and resources from a trained career coach; 3) the ability to develop a wide range of professional skills, including career confidence and leadership; and 4) the opportunity to work on achieving other career-related goals, such as increased professional advancement, job satisfaction, work–life balance, and organizational tenure. Although program recruitment did not explicitly refer to soft skill development, the curriculum did cover soft skills such as resilience, influence, and communication (*SI Appendix*, Table S7). Thus, while the TXP was geared toward a broader set of individual and interpersonal goals, our research team was most interested in examining whether this type of online program enhanced women’s overall confidence in their acquisition of soft skills, since both soft skills and confidence in one’s professional skills have been shown to predict higher workplace retention among women (1, 10, 11).

Prior to the intervention, all 44 women completed a baseline survey asking them questions related to their experiences at work, including six questions designed to measure their assessments of their soft skills.^†^ These items were averaged to create a composite measure of perceived soft skills (see Table 3 for a summary of how this measure was constructed). This measure aligns with existing definitions of soft skills used throughout the literature (1–8, 45, 46) and in practice-based settings (47).

**Table 3.** Construction of employee-perceived soft skills measure (α = 0.75)

Item	Scale
I feel capable of influencing my work colleagues.	1 = not at all; 2 = slightly; 3 = moderately; 4 = very; 5 = extremely
I feel confident in my ability to do my job well.	1 = not at all; 2 = slightly; 3 = moderately; 4 = very; 5 = extremely
I am capable of building a professional network.	1 = not at all; 2 = slightly; 3 = moderately; 4 = very; 5 = extremely
I struggle to effectively communicate my thoughts at work. (*r*)	1 = always; 2 = most of the time; 3 = about half the time; 4 = sometimes; 5 = never
I feel capable of building effective relationships with colleagues.	1 = not at all; 2 = slightly; 3 = moderately; 4 = very; 5 = extremely
I go above what is expected of me to help my team be successful.	1 = never; 2 = sometimes; 3 = about half the time; 4 = most of the time; 5 = always

Items were developed based on existing definitions of soft skills used in the literature (see refs. 3–8, 45, 46). We also drew on the Department of Labor’s soft skill curriculum (50), which aims to teach the following set of skills to young workers: communication, enthusiasm and attitude, teamwork, networking, problem solving and critical thinking, and professionalism.

We compared results from women in the intervention with those from two comparison groups who were not part of the intervention: a mixed-sex (i.e., pooled) group and a group of early-career women.^‡^ To recruit a pooled control group and a matched control group of early-career women, we circulated the same baseline survey to an additional 450 randomly selected men and women employees at the company. Of the 450 employees who received an email inviting them to participate in the study, 200 (44%) responded to the survey. We worked with the company to ensure that the control group sampling frame included an equal number of men and women employees as well as an oversampling of women who had similar characteristics to the 44 early-career women recruited for the intervention. Because early-career women made up a small proportion of the company, the treatment group in our study was composed of nearly the entire population of this cohort. Therefore, to construct a closely matched control group of early-career women, we expanded our criteria for inclusion to include women younger than 40. As a result, women in the matched control group were, on average, about 5 years older, had been with the organization for approximately 16 months longer, and received slightly higher compensation (a difference of 1.40 on an 11-point scale used by the company) than women in the treatment group. To account for these differences, our models controlled for age, employee tenure, and compensation level at baseline.

There was no random assignment to condition at this stage since participation had to be on a voluntary basis and it would have been unethical to force employees to participate. However, we confirmed with the company's analytics team that the study population within both treatment and control conditions were closely representative of early-career women at the company and the broader company population, respectively, on key demographic traits, including age, gender, tenure, and compensation level.

Since participants completed the baseline survey upon recruitment (between January and February 2020), we were able to capture soft skill self-assessments immediately prior to the COVID-19 pandemic. Upon baseline survey completion, women in the treatment group were randomly assigned to two different types of online interventions that varied only by the composition of exposure to virtual peer-group meetings and one-on-one coaching. Those assigned to the first intervention type (group A) received more exposure to peer-group meetings, whereas those assigned to the second intervention type (group B) received more exposure to one-on-one coaching. Our analytical plan consisted of two parts: 1) a comparison of the two types of interventions (group A versus group B ) and 2) a comparison of the intervention group with the two control groups, with a plan to pool the two versions of the intervention if there was no difference between them. Since we did not find any significant differences between the two types of online interventions, we pooled these two groups in our analyses into the “online intervention” condition.

For the online intervention program, we created four peer groups consisting of the same 11 members in each meeting over the course of the intervention. Prior to the first coaching or peer-group meeting (and before moving to remote work), the women attended an in-person information session at the company in February 2020. Participants were split between two women facilitators employed by the engagement platform. Each facilitator oversaw one set of 11 participants in group A and another set of 11 participants in group B to ensure that both treatment groups were exposed to the same two facilitators.

Beginning in March 2020, when nationwide lockdowns began, women participants started meeting once or twice monthly for an hour with their peer group or just the facilitator (acting as a career coach) to discuss predesignated topics. Many of these topics related to the development of professional soft skills, such as strategies for communicating with confidence and leading with influence (*SI Appendix*, Table S7). Participants set individual career goals using the online platform tools that they either reviewed with their coach or discussed in their facilitated peer groups each month. Outside of these topics, participants also had the opportunity to disclose in their peer-group meetings and coaching sessions any personal and professional challenges that they were experiencing.^§^

In July 2020, all participants were given a 3-month summer vacation hiatus from the program. The intervention resumed in October 2020. After the final peer-group meeting or coaching session in November, the women participants completed a post-intervention survey that included the exact same questions on the baseline survey. When the intervention period concluded, we distributed the same follow-up survey to all employees in the study who had taken the baseline survey but did not undergo the intervention (i.e., individuals in both control groups), collecting final responses between November 2020 and January 2021.

By the end of the study, nine women had either left the online intervention program or failed to complete the follow-up survey, leaving us with 35 women in the treatment group (80% of the original sample). Within the control group, 113 participants (56.5% of the original sample) completed the follow-up survey. However, we do not expect this attrition to meaningfully affect the interpretation of our results. When we investigated how attrition may have affected sample composition, we found that the characteristics of those who left the study and those who remained were roughly similar based on age, race, gender, tenure, performance, and compensation level within condition. Attrition in both the treatment and control groups was likely due, in large part, to a perceived lack of time. Program facilitators relayed that throughout the intervention period, which was the height of the COVID-19 pandemic, women participants reported high levels of stress and burnout affecting both themselves and employees more broadly in the organization. Some women even spoke of managers pulling them out of their peer groups or coaching sessions when work-related issues came up. Most telling, all women participants who left the intervention program cited a lack of time as justification. However, the lower rate of attrition among the treatment group compared with the control groups was likely due to the intervention program producing higher levels of study engagement.

## Supplementary Material

Supplementary File

## Data Availability

Anonymized data and analysis code have been deposited in Open Science Framework (https://osf.io/nw9md/?view_only=73a917050a174b95ac4b0d9a31cee92e) (51).

## Footnotes

^*^For a detailed classification of what constitutes soft skills in this body of literature, see refs. 4 and 5.

^†^While we focused on perceived soft skills as the primary measure of interest in the present study, we also collected an additional set of measures that were of strong interest to our TXP partner company. Information about the construction and results of these nonprimary measures is available in the *SI Appendix*, Tables S5 and S6.

^‡^See *SI Appendix*, Table S1 for an overview of baseline characteristics for the study population included in our analyses.

^§^The researchers were not involved in the creation of the program curriculum, which was proprietary to the TXP. See refs. 46, 48, and 49 for examples of other studies examining the effects of preexisting programs that target soft skills.
