# Supplementary material for: Preventing soft skill decay among early-career women in STEM during COVID-19: Evidence from a longitudinal intervention
Source: Proc Natl Acad Sci U S A. 2022 Aug 1;119(32):e2123105119. doi: 10.1073/pnas.2123105119 (PMC9371663; doi:10.1073/pnas.2123105119)
Supplement: Supplementary File [file pnas.2123105119.sapp.pdf]

PNAS
www.pnas.org

Supplementary Information for
Preventing Soft Skill Decay Among Early-Career Women in STEM During COVID-19: Evidence from a Longitudinal Intervention

Julia L. Melin, Shelley J. Correll.

Julia L. Melin
Email: jmelin@stanford.edu

This PDF file includes:

Baseline characteristics of study participant population (p. 2)
Supplemental analysis of manager assessed performance post-intervention (pp. 3-4)
Supplemental analysis examining employee perceived soft skills as a predictor of employee retention post-intervention (pp. 5-6)
Results for other non-primary survey outcome measures (pp. 7-10)
Intervention curriculum (p. 11)
SI references (p. 12)

Baseline Characteristics of Study Participant Population

Table S1 provides an overview of baseline characteristics of the study population included in our analyses. Because early-career women made up a small proportion of the company, the treatment group in our study was comprised of nearly the entire population of this cohort. Therefore, to construct a closely matched comparison group of early-career women, we expanded our criteria for inclusion to include women under the age of 40. As a result, women in the matched control group were, on average, approximately 5 years older, had been with the organization for around 16 months longer, and were compensated slightly higher (a difference of 1.40 on an 11-point scale used by the company) than women in the treatment group. To account for these differences, our models control for age, employee tenure, and compensation level at baseline.

Table S1. Baseline characteristics of study participant population (N = 148)

	**Mixed-Sex ("Pooled") Control Group**		**Matched Control Group**	**Treatment Group**
**Characteristics**	**Men (n = 46)**	**Women (n = 33)**	**Women (n = 34)**	**(n = 35)**
Age	41.47 (±10.50)	47.90 (±6.08)	33.50 (±3.90)	28.34 (±2.43)
Race, n (%)				
White	36 (78.26)	20 (60.61)	25 (73.53)	25 (71.43)
Black or African American	3 (6.52)	2 (6.06)	2 (5.88)	0 (0.00)
Hispanic or Latino	0 (0.00)	3 (9.09)	0 (0.00)	3 (8.57)
Asian	6 (13.04)	4 (12.12)	5 (14.71)	7 (20.00)
Two or More Races	0 (0.00)	1 (3.03)	0 (0.00)	0 (0.00)
Unknown	1 (2.17)	3 (9.09)	2 (5.88)	0 (0.00)
Tenure	6.21 (±7.30)	5.78 (±5.50)	3.39 (±2.51)	2.08 (±1.37)
Manager Assessed Performance, n (%) (Scale: 1-4)				
Ineligible	2 (4.35)	2 (6.06)	4 (11.76)	10 (28.57)
Does not meet expectations	2 (4.35)	0 (0.00)	1 (2.94)	1 (2.86)
Meets expectations	28 (60.87)	23 (69.70)	24 (70.59)	15 (42.86)
Exceeds expectations	12 (26.09)	7 (21.21)	4 (11.76)	6 (17.14)
Strongly exceeds expectations	2 (4.35)	1 (3.03)	1 (2.94)	3 (8.57)
Compensation Grade (Scale: 3-14)	7.06 (±2.12)	7.30 (±1.86)	6.14 (±1.74)	4.74 (±1.42)

Note: Values are presented as mean (± SD), except otherwise indicated. Ineligible indicates employees too new to the organization to receive a performance review at the time of baseline.

Supplemental Analysis of Manager Assessed Performance Post-intervention

While our study was primarily focused on evaluating the intervention's effect on self-assessments of soft skills, we also examined whether the intervention affected how managers assessed participants' overall performance. Manager's assessments of employee performance at the company consist of a single rating (scale 1-4) asking managers to reflect on an employee's overall performance over the past year across a range of both productivity-related (e.g., quality of work product, timeliness of deliverables) and interpersonal-related (e.g., leadership, collaboration) attributes. Thus, manager ratings encompass a broader range of traits and outcomes than soft skills, but do include interpersonal factors, such as collaboration.

Our sample size is reduced in this analysis due to 1) 18 employees being so new to the organization at baseline that they were ineligible to receive a performance rating (see Table S1 for reference); and 2) 8 employees leaving the company after providing post-intervention survey responses but before receiving a performance evaluation from their manager. Due to this unbalanced panel of participants, we advise some caution in the interpretation of our results. However, the below findings still provide evidence that the intervention positively affected how managers assessed the performance of early-career women in the organization throughout the pandemic.

Results. Table S2 shows results for ANCOVA estimates adjusted for baseline performance ratings, age, employee tenure, and compensation level. Model 2 shows the effect of the intervention (the reference category) on manager assessed performance, net controls, compared to the results for the matched control and pooled control groups. After adjusting for baseline differences, we found that the intervention led to significantly higher performance ratings for women in the intervention compared to those in both control groups. Compared to women who received the intervention, women in the matched control group received significantly lower performance ratings from their managers following the intervention period ($\hat{\beta}$ = -0.474, $P < 0.05$). We also observed that men and women in the pooled control group received significantly lower performance ratings compared to women in the treatment group following the intervention period ($\hat{\beta}$ = -0.464, $P < 0.05$).

Table S2. ANCOVA estimates of the effect of condition type on manager assessed performance post-intervention

	(1)	(2)
Condition Type (Ref = Online Intervention)		
Matched Control	-0.432*	-0.474*
	(0.184)	(0.187)
Pooled Control	-0.472**	-0.464*
	(0.178)	(0.203)
Manager Assessed Performance (Baseline Rating)	0.224*	0.184+
	(0.093)	(0.099)
Age		-0.007
		(0.007)
Tenure		-0.007
		(0.009)

Compensation Grade		0.053+
		(0.030)
Constant	2.213***	2.260***
	(0.261)	(0.335)
Observations	246	246
R-squared	0.154	0.176

Note: Robust standard errors in parentheses. Observations clustered at employee level. Models 1 and 2 keep individuals in the analysis who have non-missing performance data at baseline and post-intervention. Reasons for missing performance data are due to organizational attrition post-intervention and lack of performance data for individuals deemed "ineligible" at baseline.
+P < 0.10, *P < 0.05, **P < 0.01, ***P < 0.001, two-tailed tests.

Supplemental Analysis Examining Employee Perceived Soft Skills as a Predictor of Employee Retention Post-intervention

To further validate the meaningfulness of our perceived soft skills measure, we examined the relationship between perceived soft skills and organizational retention—a key concern among scholars and practitioners focused on gender inequality in STEM fields (Cech et al. 2011; Cech and Blair-Loy 2019; Ashcraft, McLain, and Eger 2016; Seron et al. 2016). Below is a summary of the results of this supplemental analysis.

Results. Table S3 shows logistic regression estimates of perceived soft skills (post-intervention) on an employee's likelihood of being employed at the company one-year post-intervention for the full sample of employee participants. The outcome variable "employed one-year post" is coded as a dummy variable, where 1 = employed one-year post-intervention and 0 = no longer employed at the company one-year post-intervention. Model 2 shows that higher perceived soft skills significantly increased an employee's odds of still being employed at the company one-year post-intervention, net controls (odds ratio = 3.21, $P < 0.01$). We also found that manager assessed performance did *not* predict employee retention (see Table S4).

Together, these findings demonstrate that employees' assessments of their own interpersonal abilities at work are more predictive of organizational retention than the assessments made by their managers. This finding is consistent with prior research showing that self-assessments around confidence and self-efficacy (at work or within a particular subject matter) are consequential for retention and persistence (Wiesenfeld et al. 2007; Judge and Hurst 2008; Seron et al. 2016)—above and beyond the effects of external evaluations of ability (Correll 2001; Cech et al. 2011)

Table S3. Logistic regression estimates of employee perceived soft skills on the likelihood of still being employed at the company one-year post-intervention (odds ratios)

	(1)	(2)
Perceived Soft Skills (Post-intervention)	2.049+	3.207**
	(0.754)	(1.341)
Age		0.912***
		(0.024)
Tenure		1.056
		(0.042)
Compensation Grade		1.225
		(0.154)
Constant	0.332	0.484
	(0.464)	(0.735)
Observations	296	296

Note: Robust standard errors in parentheses. P-value for coefficient on perceived soft skills measure in Model 1 is $p = 0.051$.
+$P < 0.10$, *$P < 0.05$, **$P < 0.01$, ***$P < 0.001$, two-tailed tests

Table S4. Logistic regression estimates of manager assessed performance on the likelihood of still being employed at the company one-year post-intervention (odds ratios)

	(1)	(2)
Manager Assessed Performance (Post-intervention Rating)	0.987	0.846
	(0.386)	(0.365)
Age		0.943+
		(0.029)
Tenure		1.021
		(0.050)
Compensation Grade		1.297+
		(0.193)
Constant	7.468*	19.483+
	(7.298)	(33.099)
Observations	280	280

Note: Robust standard errors in parentheses.
+*P* < 0.10, **P* < 0.05, ***P* < 0.01, ****P* < 0.001, two-tailed tests

Results for Other Non-primary Survey Outcome Measures

We also included other measures on the survey that related to employee experiences at work that were requested by our partner organizations. Below is a summary of these non-primary outcomes measures, how we constructed them, as well as their results from the study.

Measure Construction. Table S5 provides an overview of how we constructed the non-primary outcome measures in collaboration with our partner organizations.

Results. Table S6 shows regression estimates for results on non-primary outcome measures. Model 8 shows that compared to the treatment group, both early-career women in the matched control group and men and women in the pooled control group reported a significantly lower sense of organizational alignment ($P < 0.01$). Model 10 shows that compared to the treatment group, the matched control group reported significantly lower feelings of workplace initiative ($P < 0.05$). Model 12 shows that compared to the treatment group, the matched control group reported feeling significantly less safe asking team members for help at work ($P < 0.01$). Other models do not show significant effects.

Table S5. Non-primary outcome measure construction

Scale item measures	
Work engagement (α = .83)	How energized do you feel by the work you are doing?<br>[1 = not at all; 2 = slightly; 3 = moderately; 4 = very; 5 = extremely]<br>How often do you feel meaningfully challenged at work?<br>[1 = never; 2 = sometimes; 3 = about half the time; 4 = most of the time; 5 = always]<br>How often does your work give you a sense of personal accomplishment?<br>[1 = never; 2 = sometimes; 3 = about half the time; 4 = most of the time; 5 = always]<br>How difficult is it for you to find your work meaningful? (*r*)<br>[1 = extremely; 2 = very; 3 = moderately; 4 = slightly; 5 = not at all]
Retention intentions (α = .71)	How often do you think about quitting your job (*r*)<br>[1 = always; 2 = most of the time; 3 = about half the time; 4 = sometimes; 5 = never]<br>Will you leave your company in the next 12 months? (*r*)<br>[1 = definitely; 2 = probably; 3 = may or may not; 4 = probably not; 5 = definitely not]
Personal development (α = .80)	How much opportunity is there for growth at your company?<br>[1 = none at all; 2 = a little; 3 = a moderate amount; 4 = a lot; 5 = a great deal]<br>How often does your company make your personal development a priority?<br>[1 = never; 2 = sometimes; 3 = about half the time; 4 = most of the time; 5 = always]
Managerial relationship (α = .77)	How often does your manager give you valuable feedback to help you improve your performance?<br>[1 = never; 2 = sometimes; 3 = about half the time; 4 = most of the time; 5 = always]<br>How supported do you feel by your manager?<br>[1 = not at all; 2 = slightly; 3 = moderately; 4 = very; 5 = extremely]
Work-life balance (α = .75)	How supported are your efforts to balance work and life activities at your company?<br>[1 = not at all; 2 = slightly; 3 = moderately; 4 = very; 5 = extremely]<br>How often does your workload allow enough time for personal and family activities?<br>[1 = never; 2 = sometimes; 3 = about half the time; 4 = most of the time; 5 = always]<br>How often do you lack energy after work to do the activities you want to do? (*r*)<br>[1 = always; 2 = most of the time; 3 = about half the time; 4 = sometimes; 5 = never]<br>When you are at home (and not working), how often do you think about things going on at work? (*r*)<br>[1 = always; 2 = most of the time; 3 = about half the time; 4 = sometimes; 5 = never]

Organizational belonging (α = .82)	How connected do you feel to a community of colleagues at your company?<br>[1 = not at all; 2 = slightly; 3 = moderately; 4 = very; 5 = extremely]<br>How often do you feel like you belong at your company?<br>[1 = never; 2 = sometimes; 3 = about half the time; 4 = most of the time; 5 = always]<br>How often do you feel lonely in your organization? (*r*)<br>[1 = always; 2 = most of the time; 3 = about half the time; 4 = sometimes; 5 = never]
Single-item measures	
Overall satisfaction	Overall, how satisfied are you working at your company?<br>[1 = not at all; 2 = slightly; 3 = moderately; 4 = very; 5 = extremely]
Organizational alignment	How inspired are you by your company's vision?<br>[1 = not at all; 2 = slightly; 3 = moderately; 4 = very; 5 = extremely]
Difficult conversations	How often do you have a hard time handling difficult conversations? (*r*)<br>[1 = always; 2 = most of the time; 3 = about half the time; 4 = sometimes; 5 = never]
Work initiative	How much initiative do you take in your work?<br>[1 = none at all; 2 = a little; 3 = a moderate amount; 4 = a lot; 5 = a great deal]
Ability to make mistakes	If you make a mistake at work, how often is it held against you? (*r*)<br>[1 = always; 2 = most of the time; 3 = about half the time; 4 = sometimes; 5 = never]
Safe asking for help	How safe do you feel asking your team members for help at work?<br>[1 = not at all; 2 = slightly; 3 = moderately; 4 = very; 5 = extremely]

Note: Items assessing perceived work-life balance adapted from Schieman, Scott, Yuko K. Whitestone, and Karen Van Gundy (2006). Item assessing perceived safety asking for help adapted from Edmondson (1999). Items assessing perceived organizational belonging adapted from 3-item UCLA Loneliness Scale and the Employee Engagement Scale (ESS); see Shuck, B., Adelson, J.L. and Reio, T.G. (2017). Items assessing perceptions of work engagement, retention intentions, organizational development, organizational alignment, work initiative, and the ability to make mistakes at work are adapted from the Employee Engagement Scale (ESS); see Shuck, B., Adelson, J.L. and Reio, T.G. (2017).

Table S6. ANCOVA results for non-primary survey outcome measures

	(1) Work Engagement	(2) Personal Dev	(3) Retention Intentions	(4) Managerial Relationship	(5) Work-life balance	(6) Org belonging	(7) Overall satisfaction	(8) Org Alignment	(9) Difficult Convos	(10) Work Initiative	(11) Work Mistake	(12) Ask Help
Condition Type (Ref = Online Intervention)												
Matched Control	-0.180	-0.101	-0.0739	0.329+	-0.142	-0.0953	-0.203	-0.422**	-0.219	-0.357*	-0.060	-0.447**
	(0.122)	(0.198)	(0.124)	(0.191)	(0.153)	(0.117)	(0.153)	(0.141)	(0.151)	(0.154)	(0.164)	(0.154)
Pooled Control	-0.314+	-0.274	-0.242	0.266	-0.234	-0.187	-0.362	-0.531**	-0.311	-0.307+	0.091	-0.288
	(0.184)	(0.181)	(0.173)	(0.225)	(0.170)	(0.160)	(0.228)	(0.164)	(0.207)	(0.178)	(0.181)	(0.205)
Work engagement (baseline score)	0.613***											
	(0.088)											
Personal development (baseline score)		0.721***										
		(0.068)										
Retention intentions (baseline score)			0.851***									
			(0.066)									
Managerial relationship (baseline score)				0.510***								
				(0.080)								
Work-life balance (baseline score)					0.542***							
					(0.073)							
Organizational belonging (baseline score)						0.719***						
						(0.063)						
Overall satisfaction (baseline score)							0.659***					
							(0.072)					
Organizational alignment (baseline score)								0.595***				
								(0.076)				
Difficult conversations (baseline score)									0.428***			
									(0.073)			
Work initiative (baseline score)										0.423***		
										(0.066)		
Work mistake (baseline score)											0.625***	
											(0.069)	
Ask help (baseline score)												0.360***
												(0.082)
Age	0.022**	0.023**	0.013+	-0.005	0.024**	0.016+	0.015	0.020**	0.006	0.010	-0.002	0.030**
	(0.007)	(0.008)	(0.006)	(0.012)	(0.008)	(0.008)	(0.009)	(0.006)	(0.009)	(0.009)	(0.010)	(0.011)

Tenure	-0.011	-0.017	0.007	-0.026+	-0.005	-0.011	-0.001	-0.021**	-0.005	-0.010	0.010	-0.018
	(0.008)	(0.011)	(0.008)	(0.015)	(0.008)	(0.011)	(0.012)	(0.008)	(0.012)	(0.012)	(0.012)	(0.012)
Compensation Grade	-0.037	-0.064+	-0.012	-0.050	-0.092**	-0.050+	-0.027	0.009	0.041	0.035	-0.072*	-0.048
	(0.026)	(0.035)	(0.025)	(0.038)	(0.029)	(0.027)	(0.033)	(0.029)	(0.031)	(0.027)	(0.032)	(0.035)
Constant	1.128*	0.638*	0.178	2.143***	1.326***	0.969**	1.098*	1.353**	1.740***	2.087***	1.912***	2.040***
	(0.438)	(0.321)	(0.338)	(0.461)	(0.307)	(0.297)	(0.434)	(0.427)	(0.389)	(0.368)	(0.375)	(0.393)
Observations	294	296	296	296	296	296	294	296	296	296	296	296
R-squared	0.440	0.445	0.543	0.305	0.429	0.539	0.408	0.458	0.252	0.277	0.460	0.261

Note: Robust standard errors in parentheses; observations clustered at employee level.
+*P* < 0.10, **P* < 0.05, ***P* < 0.01, ****P* < 0.001, two-tailed tests

Intervention Curriculum

Below is a high-level overview of the content that our partner company, a talent experience platform (TXP), delivered throughout the 6-month intervention.

Table S7. Topics covered throughout 6-month intervention

**Topic**	**Description**
Sharing your journey	Reflecting on your career.
Influencing	Discovering strategies for influencing across the organization.
Negotiation	Developing negotiation skills at work.
Communication	Strategies for communicating effectively.
Goal setting	Identifying key career goals.
Communicating for impact	Defining impactful communication moments in the workplace.
Resilience	Practicing resilience and dealing with failure.
Revisiting goals	Reflecting on your journey.
Celebration	Celebrating the program experience.

SI References

1. Ashcraft, C., McLain, B., & Eger, E. Women in tech: The facts. Boulder, CO: National Center for Women and Information Technology. (2016).
2. Cech, E., Rubineau, B., Silbey, S., & Seron, C. Professional role confidence and gendered persistence in engineering. *Amer. Sociol. Rev*., **76**, 641–666 (2011).
3. Cech, E. A., & Blair-Loy, M. The changing career trajectories of new parents in STEM. *Proc. Natl. Acad. Sci*., **116**, 4182-4187 (2019).
4. Correll, S. J. Gender and the career choice process: The role of biased self-assessments. *Amer. J. Socio*., **106**,1691-1730, (2001).
5. Edmondson, A. Psychological safety and learning behavior in work teams. *Admin. Sci. Quart*. **44**, 350-383 (1999).
6. Judge, T. A., & Hurst, C. How the rich (and happy) get richer (and happier): relationship of core self-evaluations to trajectories in attaining work success. *J. Appl. Psych.,* **93***,* 849-63, (2008).
7. Schieman, S., Whitestone, Y. K., & Van, G. K. (September 01, 2006). The nature of work and the stress of higher status. *J. Health Soc. Behav.*, **47**, 3, 242-257.
8. Seron, C., Silbey, S. S., Cech, E., & Rubineau, B. Persistence is cultural: Professional socialization and the reproduction of sex segregation. *Work Occupat*., **43**, 178–214 (2016).
9. Shuck, B., Adelson, J. L., & Reio, T. G. The employee engagement scale: Initial evidence for construct validity and implications for theory and practice. *Hum. Res. Mgmt.* **56**, 953-977 (2017).
10. Wiesenfeld, B. M., Swann, Jr., W. B., Brockner, J., & Bartel, C. A. Is more fairness always preferred? Self-esteem moderates reactions to procedural justice. *Acad. Mgmt. J.*, **50**, 1235–1253, (2007).
